# A Spatial Analysis of County-level Variation in Syphilis and Gonorrhea in Guangdong Province, China

**DOI:** 10.1371/journal.pone.0019648

**Published:** 2011-05-06

**Authors:** Nicholas X. Tan, Jane P. Messina, Li-Gang Yang, Bin Yang, Michael Emch, Xiang-Sheng Chen, Myron S. Cohen, Joseph D. Tucker

**Affiliations:** 1 Harvard University, Cambridge, Massachusetts, United States of America; 2 Department of Geography, University of North Carolina, Chapel Hill, North Carolina, United States of America; 3 Guangdong Provincial STI Control Center, Guangzhou, China; 4 National Center for STD Control, Nanjing, China; 5 Division of Infectious Diseases, University of North Carolina School of Medicine, Chapel Hill, North Carolina, United States of America; University of Toronto, Canada

## Abstract

**Background:**

Sexually transmitted infections (STI) have made a resurgence in many rapidly developing regions of southern China, but there is little understanding of the social changes that contribute to this spatial distribution of STI. This study examines county-level socio-demographic characteristics associated with syphilis and gonorrhea in Guangdong Province.

**Methods/Principal Findings:**

This study uses linear regression and spatial lag regression to determine county-level (n = 97) socio-demographic characteristics associated with a greater burden of syphilis, gonorrhea, and a combined syphilis/gonorrhea index. Data were obtained from the 2005 China Population Census and published public health data. A range of socio-demographic variables including gross domestic product, the Gender Empowerment Measure, standard of living, education level, migrant population and employment are examined. Reported syphilis and gonorrhea cases are disproportionately clustered in the Pearl River Delta, the central region of Guangdong Province. A higher fraction of employed men among the adult population, higher fraction of divorced men among the adult population, and higher standard of living (based on water availability and people per room) are significantly associated with higher STI cases across all three models. Gross domestic product and gender inequality measures are not significant predictors of reported STI in these models.

**Conclusions/Significance:**

Although many ecological studies of STIs have found poverty to be associated with higher reported STI, this analysis found a greater number of reported syphilis cases in counties with a higher standard of living. Spatially targeted syphilis screening measures in regions with a higher standard of living may facilitate successful control efforts. This analysis also reinforces the importance of changing male sexual behaviors as part of a comprehensive response to syphilis control in China.

## Introduction

Although sexually transmitted infections (STIs) in China were nearly eradicated in the 1960s by massive treatment campaigns and structural measures to disrupt commercial sex, there has been a recent resurgence of reported STIs across many regions of China [1]. The reported primary syphilis cases rose from 9036 in 1996 to 40,962 in 2005 [1]. Increases in other reported sexually transmitted infections have also been noted in the last five years [2]. Since syphilis increases the risk of acquiring and transmitting HIV infection [3], [4], the increase in syphilis burden has important public health implications.

Much of the STI literature to date in China has focused on prevalence among high risk groups. Several cohort studies among men who have sex with men have found a high incidence of syphilis and sexually transmitted HIV infection [5]–[8]. High prevalence of sexually transmitted infection has been noted among female sex workers [9]–[13] and intravenous drug users [14]–[18]. A population-based representative study of chlamydia infection in China suggested that commercial sex interactions, more than casual sex, were driving chlamydia transmission [19]. Beyond cross-sectional studies of STIs in China, there have been massive social changes in China during the past twenty years that likely influenced the size and composition of groups at risk for STI.

Rapid economic development in China could expand both the size of the population of those who sell sex and the size of the population of the men who purchase it. One small ecological study found an association between municipal-level reported syphilis cases and the gross domestic product [20]. There are two mechanisms that could explain this relationship: more economically developed areas have greater “MMM”, mobile men with money, who may have higher sexual risk; or more economically developed areas have greater “surplus men”, so called because this group of unmarried, poor migrant men cannot find wives. There have been several studies analyzing wealthy businessmen in China as an important risk group for STIs [19], [21], but studies of rural-to-urban migrants in China have yielded conflicting results [22]–[26]. Some migrant studies have found that rural to urban migrants have increased sexual risk after migration, although their STI/HIV risk may not be increased compared to urban counterparts.

Developing effective public health responses to sexually transmitted infections requires a more detailed understanding of China's syphilis and gonorrhea epidemics. The distribution of STI cases is heterogeneous across China, with greater reported case burden in more economically developed provinces. Within southern China, Guangdong is the most prosperous and populous province and bears a large proportion of the national STI disease burden [1]. This study examines county-level socio-demographic characteristics associated with reported syphilis and gonorrhea in Guangdong Province. The purpose of the study was to better understand socio-demographic variables that may contribute to syphilis and gonorrhea transmission in the Chinese context.

## Methods

Guangdong Province consists of 97 county-level administrative units. The county-level is below a prefecture and above a township level, including urban districts, rural counties, and county-level cities. Various factors such as movement of migrants, economic development, and government policies have been suggested to cause income gaps, unequal infrastructure development and sexual inequality, resulting in differential social and economic conditions across counties [27], [28]. Such patterns present the unique opportunity to explore relationships between socioeconomic conditions and STI infection rates by performing spatial analysis and multiple linear regressions at the county level.

The three outcomes examined were syphilis, gonorrhea, combined syphilis and gonorrhea per 100,000 persons in 2005. All STI data was from the province-wide mandatory reporting system organized by the Guangdong Provincial Center for STI Prevention and Control and described in greater detail previously ([20]). Since there were major administrative changes to this system in 2003–2004, we only analyzed a single year following these changes. Primary and secondary syphilis cases were the focus of this analysis since these outcomes are likely more sensitive to changes in sexual behaviors compared to latent syphilis cases. The total population size figures came from the 2005 China Census [29]. A natural logarithmic transformation was used for syphilis and gonorrhea [30]. A third outcome – Combined Syphilis and Gonorrhea Index (CSGI) was created by combining syphilis and gonorrhea rates. Syphilis and gonorrhea burden was computed for each county by converting rates into standard scores according to the number of standard deviations the county observation was above or below the mean. The CSGI is the sum of equally-weighted syphilis and gonorrhea standard scores for each county.

All independent variables entered into our models are shown in Table 1. All variables except GDP and mean male income were obtained from the 2005 China Census. The total population includes both registered and unregistered population in each county. The adult population was defined as the population aged 15 and above. Variables were transformed to fit a normal distribution when appropriate. The transformations used are also shown in Table 1.

**Table 1:** Independent and Dependent Variables.

Variables *	Mean	Standard Deviation
**Syphilis rates (%)**	0.4488%	0.4512%
*Log Syphilis Rates*	−5.883559	1.048541
**Gonorrhea rates (%)**	0.8226%	0.8545%
*Log Gonorrhea Rates*	−5.46483	1.31097
*Combined Syphilis and Gonorrhea Index (CSGI)*	-	1.900347
**% population males**	50.68%	1.42%
*1/[% population males]∧3*	7.70425	0.6659999
**% adult population married**	68.28%	5.63%
[% adult population married]∧3	0.3303608	0.0703867
**% adult population divorced males**	0.42%	0.18%
*Log [% adult population divorced males]*	−5.569703	0.4334807
**% adult population divorced females**	0.29%	0.26%
Log [% adult population divorced females]	−6.220428	0.9376379
**% population aged 20–40 year old female**	16.14%	4.71%
1/[% population aged 20–40 year old female]	6.614158	1.535347
**% population aged 40–60**	24.05%	4.44%
*[% population aged 40–60]∧2*	0.0614522	0.0194678
**% population unregistered**	20.30%	20.94%
1/Square Root [% population unregistered]	3.150303	1.45058
**% adult population with Junior College Education and above**	5.54%	6.23%
*Log [% adult population with Junior College Education and above]*	−3.300464	0.8766884
**% adult population employed males**	49.47%	1.66%
*1/square root [% adult population employed males]*	1.708146	0.1012627
**20–24 year old female fertility rate (%)**	10808.28%	5837.62%
*Square Root [20–24 year old female fertility rate]*	10.01813	2.790003
**Illiteracy Rate (%)**	4.34%	2.96%
*Log [Illiteracy Rate]*	−3.396999	0.7743066
**GDP per capita (US$/year)**	278.3324	421.2602
*Log GDP*	4.860471	1.192134
**Average Male Income (US$/year)**	4890.595	7117.137
*1/Average Male Income*	0.0004131	0.0002531
**Gender Empowerment Measure**	0.4652261	0.1123762
**Standard Of Living Index**	-	1.031737

*Bold denotes untransformed variables.

When the values for a sociodemographic variable were available separately for males and females, they were first tested for correlation and multicollinearity in a preliminary Ordinary Least Squares (OLS) regression analysis. Both male and female variables were evaluated separately only if there was no multicollinearity problem. If the separate male and female variables were highly correlated and/or led to multicollinearity, then these variables were either combined into a single variable encompassing both sexes, or one of the variables (male or female) was dropped. The proportion of the adult population that was married, percentage aged 40–60, percentage unregistered, and percentage of adults with a minimum junior college education were combined. Females aged 20–40 and percentage of males employed were used.

Illiteracy rates were reweighted to penalize differences in literacy achievement between males and females. They were equally distributed by sex using an equation that accounts for sexual disparities (Text S1). This technique of accounting sexual disparities has been recommended by the United Nations Human Development Report Guidelines [30]. Average male income was based on a specific method laid out by the United Nations Human Development Report Guidelines (Text S1). The Gender Empowerment Measure (GEM) is an indicator of male and female influence in the political and economic arena. It was first introduced in the Human Development Report 1995 to measure human development with an emphasis on highlighting female status [30]. This is important because women's rights in China have been an issue for a long time [31], and their oppression has been suggested to contribute to the recent syphilis epidemic. GEM comprises three components: parliamentary representation, economic participation, and income. Parliamentary representation was defined as all positions in public administration, international and social organizations. Economic participation was defined by the number of managerial, administrator and professional level positions. Average male and female income was calculated in the same manner as described earlier. The parliamentary equally distributed equivalent percentage (EDEP) and economic EDEP were calculated using the formula specified in Text S1. The Standard of Living Index was computed by summing the standard scores of the availability of safe water and the average number of rooms per person, as suggested by the United Nations Millennium Goals Indicators [31].

A Spearman's Rank Correlation was first calculated between all independent and dependent variables to examine individual associations between the socioeconomic variables and STI incidence rates. Although this provided an indication of the correlation between variables and STI incidence, all variables were entered into the multiple linear regression models. A series of backward stepwise regression was performed for each dependent variable starting with all the independent variables. The probability cut-off value for retention in the model was set at 0.05. Finally, spatial lag regression analyses were conducted by entering the significant socioeconomic variables from the linear regression models. Geographic Information System files for the 97 counties were obtained from the Harvard Geospatial Library [32]. Previous research has shown that syphilis and gonorrhea cases cluster geographically; hence accounting for spatial autocorrelation may provide more accurate estimates for the proximal determinants of STIs. Spatial lag regression considers not only the values of the dependent variables, but also the values observed in surrounding counties as defined by a spatial weighting matrix. We used SAS version 9.2 for the univariate, bivariate, and multiple linear regression analyses and GeoDa version 0.9.8.14 for the spatial regression analyses.

## Results

The mean and standard deviations of independent and dependant variables are shown in Table 1. The mean value for gonorrhea rates was 0.8% while syphilis rates had a mean of 0.4%. Table 2 displays the findings from the bivariate Spearman's rank correlation analysis. Syphilis incidence rates and CSGI analyses yielded the same results. Syphilis incidence and CSGI had a strong positive correlation with divorced females, females aged 20–40, unregistered population, population with education above junior college level, average male income, and gender empowerment measure (*r*>*0.5*); and strong negative correlation with illiteracy rates (*r*<*−0.5*). Gonorrhea incidence had strong positive correlation with divorced females, females aged 20–40, unregistered population, population with education above junior college level, average male income, and gender empowerment measure (*r*>*0.5*); and strong negative correlation with illiteracy rates and 20–24 year old fertility rates (*r*<*−0.5*).

**Table 2:** Spearman's Rank Correlation.

	Syphilis	Gonorrhea	CSGI
% population males	0.0309	0.0288	0.0313
% population married	0.049	−0.041	0.0052
% population divorced males	0.3151	0.1526	0.2433
% population divorced females	0.7333	0.7423	0.7833
% population aged 20–40 year old female	0.5649	0.674	0.6582
% population aged 40–60	0.0059	−0.0266	−0.0066
% population unregistered	0.637	0.7006	0.7141
% adult population with Junior College Education and above	0.7038	0.6728	0.7271
% adult population employed males	0.1604	0.2697	0.2185
20–24 year old female fertility rate (%)	−0.3787	−0.5214	−0.4794
Illiteracy Rate (%)	−0.5568	−0.6786	−0.6555
GDP per capita	0.3038	0.4019	0.3689
Average Male Income (US$/year)	0.5251	0.5991	0.5975
Gender Empowerment Measure	0.5336	0.5618	0.5878
Standard Of Living Index	0.4233	0.4486	0.4672

**Figure 1:**
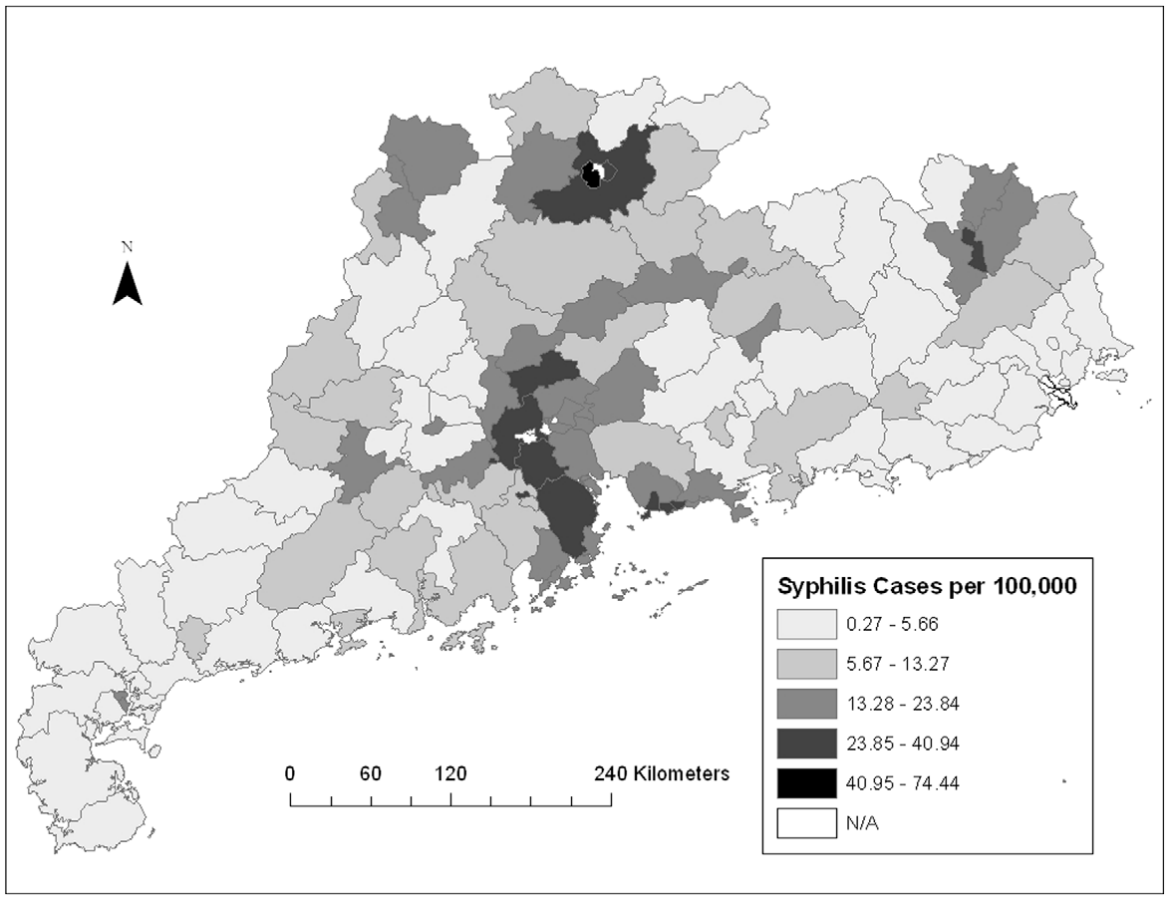
Spatial distribution of syphilis cases per 100,000 adults in Guangdong Province. This figure shows that although syphilis burden is not clustered across the entire Guangdong province, there is higher syphilis burden in the counties in the central region, called the Pearl River Delta.

**Figure 2:**
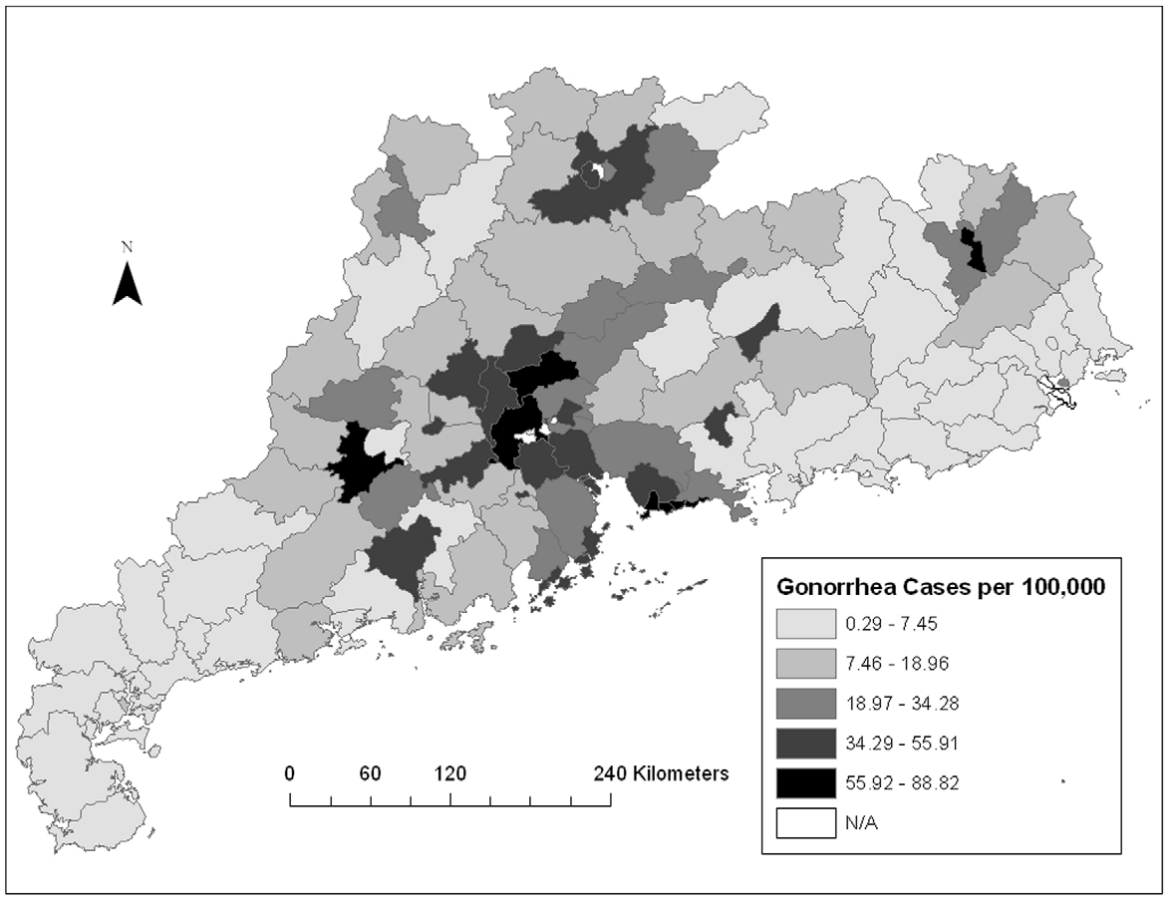
Spatial distribution of gonorrhea cases per 100,000 adults in Guangdong Province. This figure shows a similar clustering pattern to syphilis. There is higher gonorrhea burden in the counties in the central region of Guangdong Province.

The map showing the distribution of syphilis cases showed one cluster of high incidence rates in the central region of Guangdong Province and two isolated clusters of low incidence rates in the southeastern and western counties (Figure 1). The distribution of gonorrhea cases showed a similar clustering pattern (Figure 2). Univariate LISA cluster maps for both syphilis and gonorrhea were also created (Figures S1 and S2). Syphilis and gonorrhea cases showed overall weak spatial association with the presence of a few isolated clusters.

The results of the backward stepwise OLS regression for syphilis, gonorrhea, and CSGI are displayed in Table 3. OLS regression analysis showed that higher syphilis incidence rates at county level were significantly associated with higher percentages of employed males, divorced males, higher standards of living, unregistered population, and 20–40 year old females. The OLS model had an adjusted R^2^ of 66.5%. Other sociodemographic variables were eliminated in the following order – 20–24 year old fertility rates, illiteracy rates, average male income, divorced females, GEM, GDP, population aged 40–60, married population, population with education above junior college level, and percentage of total males.

**Table 3:** Backward Stepwise Regression and Spatial Lag Regression models.

	Backward Stepwise Regression (n = 97)			Spatial Lag Regression (n = 97)		
	Unstandardized Coefficient	Standard Error	P-Value	Unstandardized Coefficient	Standard Error	P Value
**Syphilis**						
*% adult population employed males* *	−2.016042	0.7411878	0.00782	−1.870957	0.722857	0.00965
% population unregistered*	−0.2168563	0.06814441	0.00200	−0.2089737	0.06630818	0.00162
*% population divorced males*	1.40436	0.1703864	0.00000	1.359753	0.1663898	0.00000
Standard Of Living Index	0.3153882	0.07262007	0.00004	0.3085046	0.07063666	0.00001
% population aged 20–40 year old female*	−0.2109721	0.06410162	0.00142	−0.2058472	0.062106	0.00092
Adjusted R squared	0.665383			0.685503		
Spatial Parameter	-			0.06211572	0.06435821	0.33447
Akaike Information Criterion	182.774			183.987		
Log likelihood	−85.3871			−84.9935		
**Gonorrhea**						
*% adult population employed males* *	−3.893117	0.7919251	0	−3.750062	0.7856825	0
Illiteracy Rate	−0.3450462	0.1250601	0.0070083	−0.3425638	0.1215131	0.0048152
*% population divorced males*	0.6118275	0.2212174	0.0068773	0.6097434	0.2139931	0.0043809
Standard Of Living Index	0.3267239	0.08492692	0.0002215	0.3204018	0.08245565	0.0001021
*% population divorced females*	0.6076922	0.1062824	0	0.5840591	0.1077762	0
Adjusted R squared	0.682448			0.700008		
Spatial Parameter				0.04291393	0.07238207	0.5532609
Akaike Information Criterion	206.055			207.744000		
Log likelihood	−97.0275			−96.872100		
**Combined Syphilis and Gonorrhea Index (CSGI)**						
*% adult population employed males* *	−5.885109	1.127263	0	−4.576749	1.109204	0
*% population divorced males*	1.452001	0.3225491	0	1.273677	0.3052852	0
*% population divorced females*	0.7257388	0.1781957	0	0.5770036	0.1709695	0.0007386
Standard Of Living Index	0.5815712	0.1176782	0	0.5326644	0.1112608	0
% population unregistered*	−0.3482218	0.1114422	0.0023884	−0.3079282	0.1067888	0.0039326
Adjusted R squared	0.71437			0.747618		
Spatial Parameter				0.241017	0.08202854	0.0033013
Akaike Information Criterion	275.639			271.411		
Log likelihood	−131.82			−128.705		

*Inversely transformed variables.

The probability values of the independent variables in the spatial lag regression model were similar to the OLS model (p<0.01) and their relative coefficients remained almost the same. The spatial lag coefficient was insignificant. R^2^ showed little improvement from 66.5% to 68.6%. Akaike's information criterion (AIC) and log likelihood for the spatial lag model were higher which meant that there was no statistical improvement in the spatial lag model.

OLS regression showed that higher gonorrhea rates at county level were significantly associated with higher percentages of employed males, divorced males, divorced females, and higher standard of living. Higher gonorrhea rates were also associated with lower illiteracy rates. The OLS model had an adjusted R^2^ of 68.2%.

Similar to the syphilis models, the probability values of the independent variables in the spatial lag regression model were similar to the OLS model (p<0.01) and their relative coefficients remained almost the same. The spatial lag coefficient was insignificant. R^2^ had little improvement from 68.2% to 70.0%. AIC and log likelihood for the spatial lag model were higher too which meant that there was no statistical improvement in the spatial lag model.

When combined, the OLS model for CSGI was very similar to the OLS for gonorrhea incidence rates. Higher CSGI at county level were significantly associated with higher percentages of employed males, divorced males, divorced females, standard of living, total males, and unregistered population. Higher CSGI was also strongly associated with lower percentage of population aged 40–60. This OLS model had had an adjusted R^2^ of 74.3%.Other sociodemographic variables were eliminated in the following order: 20–24 year old fertility rates, average male income, GDP, illiteracy rates, population with education above junior college level, married population, females aged 20–40, and GEM.

The results of the independent variables in the spatial lag regression model were similar to the OLS model (p<0.02) and their relative coefficients remained almost the same. However, the spatial lag coefficient was significant (p = 0.03). R^2^ showed improvement from 74.3% to 77.1%. AIC and log likelihood for the spatial lag model was lower which meant that the spatial lag model provided a better fit.

## Discussion

Syphilis has become a major public health scourge in many regions of southern China. During 2008, there were more reported syphilis cases in Guangdong Province than the entire European Union, highlighting the importance of better understanding the re-emergence of syphilis [2]. The dramatic spatial variation in regional syphilis burden largely tracks along pathways of economic development. Using a spatial analytical approach, this study discovered several regions of Guangdong Province with a higher burden of reported syphilis and gonorrhea (Figure 1), consistent with other spatial analyses of these STIs [33]–[36]. This is the first spatial analytical study that reports primary and secondary syphilis cases in China. Most of our understanding of syphilis epidemiology has come from small cross-sectional studies of high-risk groups or routine screening among pregnant women [26], [37], [38]. These studies identified a high prevalence of syphilis, but could not be used to better understand the larger social determinants driving syphilis transmission.

Three sociodemographic factors were significantly associated with syphilis, gonorrhea, and the combined index in all three models: higher fraction of employed men among the adult population, higher fraction of divorced men among the adult population, and higher standard of living.

The finding that greater numbers of divorced men are associated with higher STI burden is contrary to a United States-based ecological study that found no association [39]. This could be due to higher divorce rates among Chinese men who have sex with men (MSM), greater numbers of partners among those who are divorced, or syphilis contributing to divorce. Approximately one-third of MSM in China are married to a woman [38], [40]. Unhappy or unsatisfied MSM who eventually become divorced report more unprotected sex and have a higher risk of STIs [41]. STI transmission from MSM to their wives could have a substantial impact on STI transmission in the general Chinese population, although there are not yet empirical studies reflecting this trend [42], [43]. Another possible explanation is that divorced individuals tend to have more sexual partners than single or married individuals. Divorced women on average have more lifetime sexual partners compared to non-married women [44]. In addition, syphilis and associated unsafe sex might contribute to a marriage breaking up, contributing to the observed association.

A higher fraction of employed men among the adult population was positively associated with higher reported STI cases. This finding is consistent with earlier reports with increased STI risk among employed Chinese men [19], [21]. The availability of a disposable income allows them to purchase sex and potentially pay more for not using a condom [21], fueling the commercial sex industry while concomitantly transmitting STI. A higher share of employed men associated with syphilis and gonorrhea could also be related to group norms established in work settings or to work-related trips to purchase sex [21].

The percentage of unregistered population was significant in two of the three models and substantially varied according to STI burden. Rural to urban Chinese migrants are often drawn by better employment opportunities in urban regions, but following their arrival in urban areas may have different sexual norms and limited STI knowledge [45], [46]. The literature on rural-to-urban migrant STI/HIV risk has reflected the great heterogeneity in this migrant population [47]–[49].

A higher standard of living was associated with higher STI infection rates across all three models while GDP and average male income were not significant. Another study from Guangdong found that GDP was significantly associated with reported syphilis cases at the municipal level, but did not account for other socio-demographic variables [50]. Poverty is usually negatively correlated with STI rates [51]–[53]; however positive correlations have been reported before [54]–[57].

Gender inequalities have been suggested as an important driver of STI transmission in many contexts [58]–[61]. Women who have fewer economic opportunities may have a greater chance of selling unsafe sex [58], [62]. However, gender Empowerment Measure (GEM) which measures male and female opportunities in economic and political arenas was not a significant predictor of STI infection.

There are several limitations to this study. First, this study performed analysis and drew conclusions from 2005 syphilis and sociodemographic data. We did not perform analysis on a more recent year because more complete sociodemographic data were available from 2005. Second, the county where an individual becomes infected with STI might not be the same as the county where he or she seeks treatment, either due to travelling or to stigma associated with seeking STI services. Third, there are many STI infections that go untested or unreported. A population-representative survey from China found that only 40% patients with symptoms seek medical services [19]. Fourth, this study only included 97 counties, limiting the ability to generalize to other provinces or regions. Finally, although this analysis focused on the county level because this was the most granular level available, policies focused on sexual health promotion are more frequently instituted at higher levels (e.g., municipality, province).

This research has two important implications for syphilis control programs in China related to identifying high risk geographies and high risk groups. The finding that counties with a higher standard of living have a greater burden of syphilis infection can inform spatially-targeted control efforts. Spatially focused syphilis control programs have been suggested in China and abroad to target campaigns where they are most needed [63]. Furthermore, this research study helps us to infer about heterosexual men at increased risk of syphilis. This group of individuals is poorly represented in the literature and difficult to reach using conventional surveillance and epidemiological research techniques [64].

The new ten-year plan for syphilis control includes expansion of syphilis testing at HIV voluntary counseling and testing (VCT) clinics, methadone maintenance clinics, and among certain typologies of female sex worker. Current syphilis intervention programs and research target mostly female risk groups, but this spatial analysis highlights the need to better identify and intervene on behalf of high-risk men. The large demographic changes underway in China will change sex ratios and may influence sexual norms and sexual behaviors. Further research is needed to understand the rapid increase in reported syphilis burden and target resources for intervention.

## Supporting Information

Text S1 Formulas used to calculate potential predictor variables.
(DOC)

Figure S1 Univariate LISA cluster map of syphilis cases in Guangdong Province.
(TIFF)

Figure S2 Univariate LISA cluster map of gonorrhea cases in Guangdong Province.
(TIFF)
