# Supplementary material for: A Spatial Analysis of County-level Variation in Syphilis and Gonorrhea in Guangdong Province, China
Source: PLoS One. 2011 May 6;6(5):e19648. doi: 10.1371/journal.pone.0019648 (PMC3089632; doi:10.1371/journal.pone.0019648)

**Text S1. Formulas used to calculate potential predictor variables.**


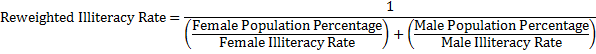


The female share of the wage bill was calculated according to the following formula:


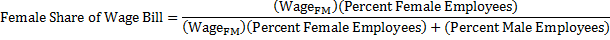


Wage_FM_ was the ratio of female to male wage. Since there were no publicly available figures on this ratio, a value of 75% was used as recommended. Percent Female Employees and Percent Male Employees were the number of employed females and males divided by the total employed population respectively. They represent the male and female shares of the economically active population.

Next, the average male income was calculated according to the following formula:


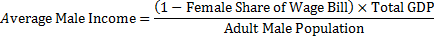


The Gender Empowerment Measure (GEM) was calculated by the following formulas:


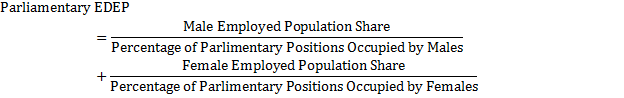


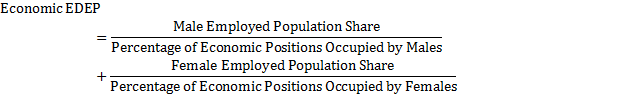

Supplement: Text S1 — Formulas used to calculate potential predictor variables. (DOC) [file pone.0019648.s001.doc]
