# Supplementary figures and images for: A Spatial Analysis of County-level Variation in Syphilis and Gonorrhea in Guangdong Province, China
Source: PLoS One. 2011 May 6;6(5):e19648. doi: 10.1371/journal.pone.0019648 (PMC3089632; doi:10.1371/journal.pone.0019648)

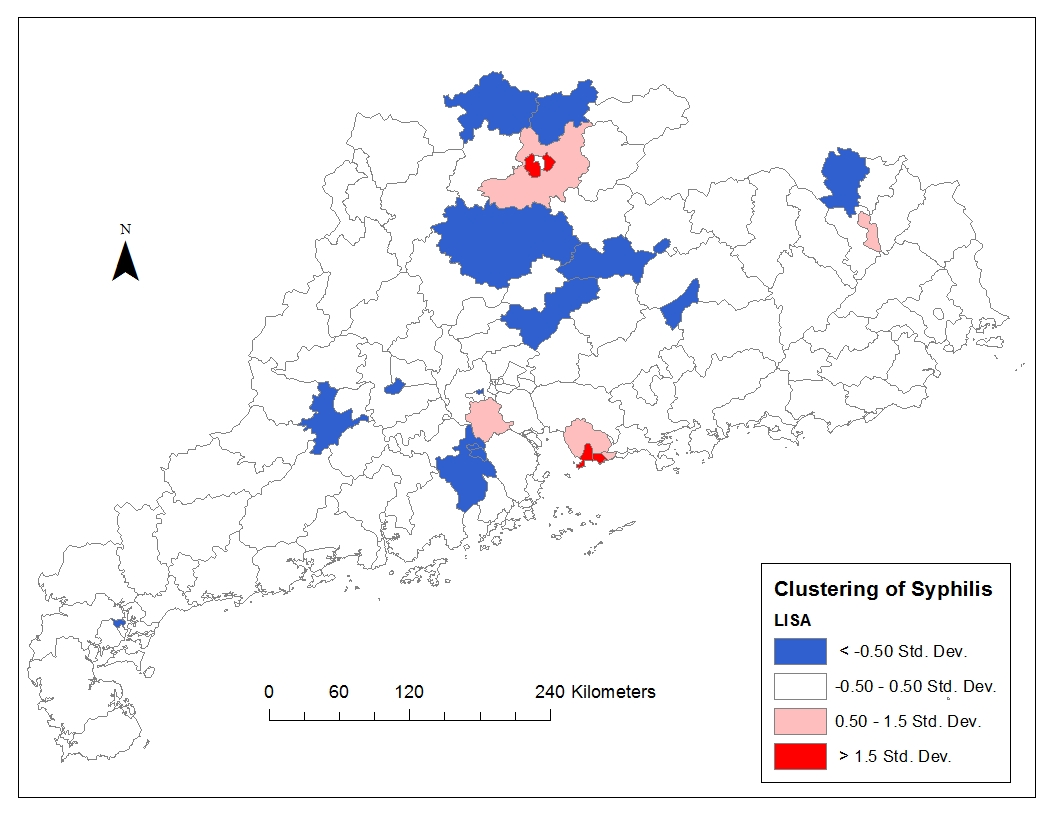

Supplement: Figure S1 — Univariate LISA cluster map of syphilis cases in Guangdong Province. (TIFF) [file pone.0019648.s002.tif]

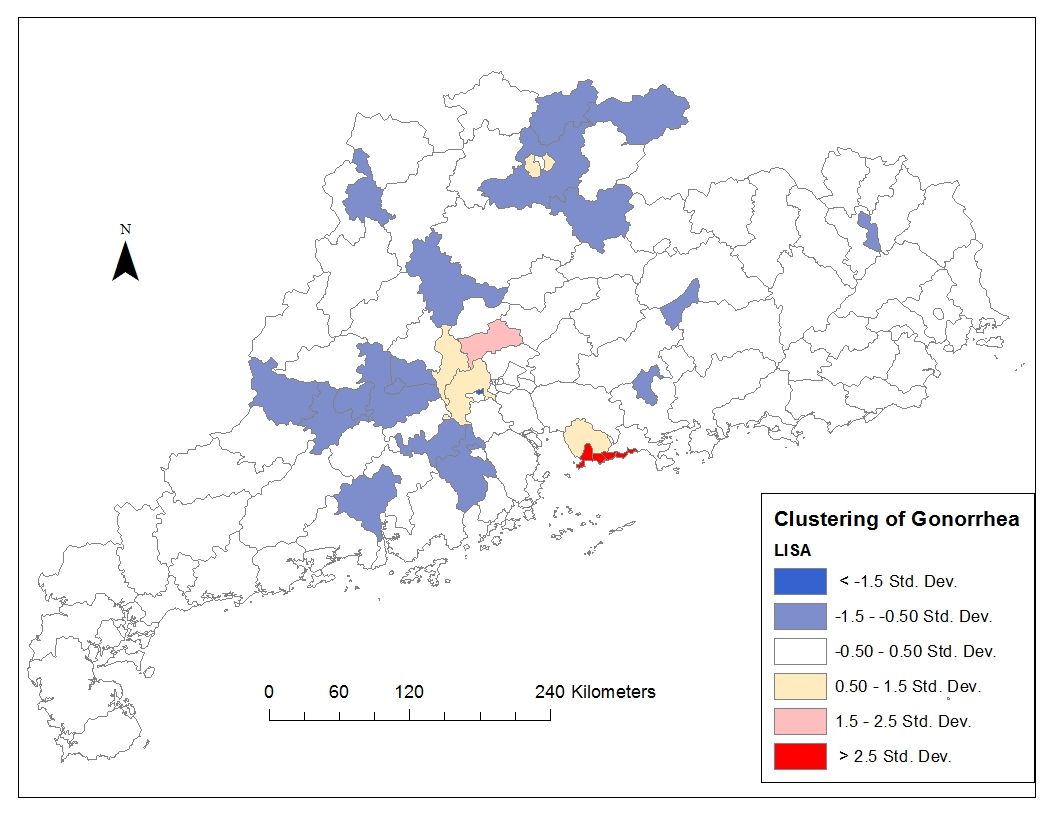

Supplement: Figure S2 — Univariate LISA cluster map of gonorrhea cases in Guangdong Province. (TIFF) [file pone.0019648.s003.tif]
